# Temporal dynamics of sapota pest damage and *Phytophthora* disease: insights from time series and machine learning models

**DOI:** 10.3389/fpls.2025.1659709

**Published:** 2025-09-23

**Authors:** Meenakshi Malik, Niranjan Singh, Amoghavarsha Chittaragi, Raghavendra D, Balanagouda Patil, Bachu Lakshmi Manisha

**Affiliations:** ^1^ Agricultural Statistics, Indian Council of Agricultural Research (ICAR)-National Research Institute for Integrated Pest Management, New Delhi, India; ^2^ Computer Applications, ICAR-National Research Institute for Integrated Pest Management, New Delhi, India; ^3^ ICAR-KVK, Chintamani, University of Agricultural Sciences, GKVK, Bangalore, India; ^4^ Entomology, ICAR-National Research Institute for Integrated Pest Management, New Delhi, India; ^5^ Plant Pathology, Keladi Shivappa Nayaka University of Agricultural and Horticultural Sciences, Iruvakki, Sagar, India

**Keywords:** sapota, pest damage, phytophthora disease, climatic factors, ARIMA, SARIMA, VAR, random forest

## Abstract

**Introduction:**

Sapota (*Manilkara zapota* L.) is a major tropical fruit crop prone to damage by bud borer (*Anarsia achrasella*), seed borer (*Trymalitis margarias*), and fruit rot caused by *Phytophthora* species. Climatic variability strongly influences these biotic stresses, yet long-term temporal patterns remain poorly quantified.

**Methods:**

A decade-long dataset (2014–2022) from 21 major sapota-growing districts of Maharashtra, India, was analyzed to study pest and disease dynamics. Statistical and machine learning approaches, including ARIMA, SARIMA, and VAR time-series models, along with Random Forest feature importance analysis, were applied to quantify climatic influences and forecast severity trends. Correlation analyses were used to assess weather–pest/disease associations.

**Results:**

Trend analysis revealed fluctuating bud and seed borer damage, while *Phytophthora* disease severity remained relatively stable. Bud borer incidence was positively correlated with rainfall (r = 0.69), seed borer with maximum temperature (r = 0.47), and *Phytophthora* with minimum temperature (r = 0.64). The ARIMA model provided accurate forecasts for bud borer (MSE = 8.03) and *Phytophthora* (MSE = 0.20), while the VAR model performed best for seed borer (MSE = 17.96). Random Forest analysis identified minimum temperature as the most critical driver of bud borer and *Phytophthora* severity, whereas relative humidity was most influential for seed borer.

**Discussion:**

The integration of statistical and machine learning models provides robust insights into sapota pest and disease epidemiology under climatic variability. These findings highlight the importance of temperature, humidity, and rainfall in shaping pest–pathogen interactions and provide predictive tools to design timely, targeted, and climate-resilient management strategies for sapota cultivation.

## 1 Introduction

Sapota (*Manilkara zapota* L.) is a prominent tropical fruit crop belonging to the Sapotaceae family is widely cultivated in various tropical and subtropical regions globally, including India, Mexico, Philippines and other Southeast Asian countries. India ranks among the top global producers of sapota, with approximately 170,000 hectares under cultivation and an annual production of 1.3 million tonnes (ICAR, 2021). Sapota offers various nutritional benefits being rich in vitamins, minerals, dietary fiber and possesses pharmacological properties including antioxidant and anti-inflammatory effects (Kaur et al., 2020). However, the productivity remains sub-optimal due to recurring pest and disease outbreaks.

The cultivation of sapota is highly susceptible to a range of pests and diseases which can significantly impact yield and quality. Numerous pests including the Sapota bud borer (*Anarsia achrasella* Bradley), Mealybug (*Planococcus citri* Risso), Leaf webber (*Nephopteryx eugraphella* Ragonot), Scale insects (*Pulvinaria psidii* Maskell) and Thrips (*Scirtothrips dorsalis* Hood) are known to cause substantial economic losses (Mani and Jayanthi, 2022; Sathish et al., 2014). These pests not only reduce fruit quality and marketability but also lead to significant declines in production due to their direct damage and indirect effects on plant health. Among the most damaging are the sapota bud borer, Sapota seed borer and disease caused by *Phytophthora* species. Yield losses due to sapota bud borer have been estimated at 30–40%, particularly during peak flowering and fruit setting periods (Mani and Jayanthi, 2022). Seed borer can cause 20–25% fruit loss, rendering the fruits unmarketable (Bisane, 2016), while Phytophthora fruit rot leads to 10–20% loss under favorable climatic conditions, with even higher losses (>50%) reported during years with heavy monsoon rains and poor drainage (Malshe and Shinde, 2016). These biotic stresses contribute to substantial economic losses annually, adversely affecting farmer income and supply chains.

The sapota bud borer is a notorious insect pest that primarily targets young shoots and flower buds, leading to considerable yield losses (Mani and Jayanthi, 2022). The larvae bore into the buds, causing wilting, drying and eventual dropping of the affected plant parts thereby directly impacting fruit setting and overall productivity which is a significant threat to fruit development and marketability whereas the sapota seed borer primarily targets the developing seeds within the sapota fruit, leading to considerable yield losses and rendering affected fruits unmarketable (Mani and Jayanthi, 2022). The larvae bore into the fruit and feed on the seeds, causing internal damage that can lead to premature fruit drop, rotting and reduced fruit quality. This direct damage to the fruit’s core significantly impacts overall productivity and the economic viability of sapota cultivation.

Concurrently, *Phytophthora* species pose a severe threat to sapota cultivation, causing various symptoms such as root rot, collar rot, and fruit rot (Joshi et al., 2014; Somwanshi et al., 2021). These pathogens are particularly destructive in hot and humid tropical environments where sapota is extensively cultivated. *Phytophthora* infections typically manifest as water-soaked lesions on the affected tissues, leading to rapid decay and plant mortality in severe cases. *Phytophthora* can lead to fruit rot, especially on fruits in the lower canopy. Its incidence is highly dependent on environmental conditions with heavy rain during the monsoon season and poor drainage exacerbating disease severity. This pathogen thrives in conditions of elevated temperatures and high humidity, particularly when water directly contacts the fruit (Liu et al., 2018; Joshi et al., 2014). The persistent and rapid spread of *Phytophthora* diseases, thriving under favorable weather conditions, has made them a significant challenge and threat to sapota growers (Joshi et al., 2014).

High humidity and extended periods of rainfall create ideal conditions for the proliferation and spread of *Phytophthora* especially during and after monsoon seasons similarly, minimum temperature and low rainfall, relative humidity can favor the life cycle and population dynamics of the sapota bud borer (Gajera et al., 2023) likewise low rainfall, high relative humidity and bright sunshine hours lead to higher infestations of sapota seed borer (Bisane and Naik, 2021). These shifts in temperature and other atmospheric conditions can markedly alter the prevalence of pest status. This is attributed to their direct bearing on the viability, propagation and spread of insect pests and pathogens alike. Hence, a holistic grasp of the complex interplay and the intricate connections among climatic elements, pest ethology and disease progression becomes paramount significance for establishing focused and efficacious management protocols in sapota cultivation (Iglesias and Rosenzweig, 2007; Estay et al., 2014).

Though previous research consistently shows that global climate change exacerbates the risk of increased pest incidence in agricultural regions (Elad and Pertot, 2014.) and describe the influence of weather parameters on population of sapota bud borer, seed borer and *Phytophthora* disease, studies on long-term seasonal trends of how these pattern change over time are to be emphasized upon which the overall pattern of pest migration strategy and sudden pest outbreaks can be predicted (Thumar Rasiklal et al., 2015). Using time series models ARIMA (Autoregressive Integrated Moving Average) and SARIMA (Seasonal Autoregressive Integrated Moving Average) can give us a much better scope to understand these patterns with minute detailing, as they can capture both quick changes and long-term seasonal trends in assessing the severity of pests and diseases (Box et al., 2015; Loona et al., 2025; Patil et al., 2025; Singh et al., 2025).

Accurate forecasting of pest and disease dynamics also holds immense practical relevance by facilitating time-sensitive and need-based insecticide and fungicide applications (Loona et al., 2025). By anticipating the peak periods of bud borer and seed borer infestation, or the onset of Phytophthora outbreaks, farmers can align chemical interventions more precisely with pest and pathogen activity windows. This minimizes the risk of premature or delayed sprays, both of which reduce efficacy and increase costs. Early warnings derived from ARIMA and SARIMA models can help optimize the timing and frequency of pesticide applications, enhancing control efficiency while reducing unnecessary chemical use (Loona et al., 2025; Patil et al., 2025; Singh et al., 2025). Such targeted application not only mitigates yield losses and economic damage but also reduces environmental contamination and pesticide residues in agriculture (Amoghavarsha et al., 2021). Integrating predictive modeling into pest management schedules thus serves as a crucial tool for advancing sustainable sapota cultivation under increasingly variable climatic conditions.

Present study directly addresses key research gaps by investigating the severity of both sapota bud borer seed borer and *Phytophthora* across various agro-climatic regions. We aim to develop and validate ARIMA and SARIMA forecasting models to accurately predict the incidence and provide precise forewarnings, enabling farmers to implement timely and effective management strategies. Furthermore, our research will emphasize the critical need to integrate climatic data into agricultural planning, boosting sapota cultivation’s resilience against the increasing variability in weather patterns caused by climate change. Ultimately, the findings from this study are expected to empower farmers and policymakers to propose targeted pest and disease management strategies.

## 2 Materials and methods

### 2.1 Study area and data collection

The study was conducted to analyze pest damage and disease severity in sapota (Manilkara zapota (L.) P. Royen) cultivation across 21 major sapota-producing districts of Maharashtra, India, from January 2014 to December 2022. These districts—covering the Vidarbha, Marathwada, and Western Maharashtra agro-climatic zones—include Jalna, Wardha, Amravati, Nanded, Aurangabad, Akola, Chandrapur, Jalgaon, Buldana, Yavatmal, Ahmadnagar, Dhule, Nagpur, Nandurbar, Nashik, Parbhani, Beed, Gadchiroli, Hingoli, Washim, and Chhatrapati Sambhajinagar.

Guidelines for scouting of pests in sapota include selection of orchards and trees, wherein randomly one village may be covered in the morning and another in the evening. In each orchard, 4 trees were observed by selecting one tree from each direction (E, S, W and N). Orchards having at least one acre area and assigned and villages at 10 Km distance are preferred, however, adjoining village was also considered if it has at least 50 ha area. The names of village and number of growers were noted for fixed plots only.

Method of observations of Insects: **Sapota bud borer**: The number of buds infested due to the pest and total number of buds on ten shoot in each direction i.e. E, S, W and N of the tree were recorded weekly and four trees in each of the selected orchard were observed for noting the pest population, and further identification was made based on the symptoms of damage made by the pest, in this manner total number of shoots observed per orchard were 160. Per cent bud damage was thus calculated. **Sapota seed borer**: The total number of harvested fruits on the daily basis from the orchard and from the total harvested fruits were record the number of fruits damaged due to the seed borer were also documented. Addition of the data for all the five days and recording it into the data sheet on was carried out on Saturday. Also two light traps per ha are installed in the selected orchards and weekly count of the seed borer trapped in each trap was taken. ***Phytophthora disease***: 10 shoots in each direction of the tree were observed for the disease incience. Grading the disease intensity on each shoot was done the scale of 0-4 as follow [see 01.png]

Rating Scale:
0 = No. incidence.
1 =1 - 20% incidence
2 = 21 - 40% incidence
3 = 41 - 60% incidence
4 = 61 -100% incidence

Percent disease incidence will be calculated by following formula.

Each orchard acted as a replication, with monthly data averaged across five trees per site, resulting in robust biological replication and within-location consistency. Standard pest and disease assessment protocols were followed across all years and districts. To ensure uniformity, field staff underwent annual training, and standardized proforma were used. Geo-referencing was done for each orchard, and efforts were made to conduct assessments at consistent time windows each month, minimizing observational bias.

Meteorological data, including daily maximum and minimum temperature, relative humidity, and rainfall, were obtained from India Meteorological Department (IMD) stations located nearest to each sampling district. These data were aggregated into monthly means to align with the pest and disease observations.

### 2.2 Correlation analysis

Correlation analysis was conducted to explore the relationship between pest damage, disease severity, and weather variables. The Pearson correlation coefficient was used to determine the strength and direction of the relationship between each pest or disease variable and weather parameters, such as maximum temperature, minimum temperature, relative humidity, and rainfall.

### 2.3 Time series analysis

To forecast future trends in pest damage and disease severity, three different time series models were employed: ARIMA (Auto Regressive Integrated Moving Average), SARIMA (Seasonal Auto Regressive Integrated Moving Average), and VAR (Vector Autoregressive). To examine the temporal stability of the residuals and detect any potential structural breaks in the time series data, the CUSUM (Cumulative Sum) test was performed on the residuals of each forecast model. The residuals were plotted against critical bounds to assess the constancy of parameters over time.

#### 2.3.1 ARIMA model

The ARIMA (1, 1, and 1) model was applied to the time series data for Bud Borer Damage, Seed Borer Damage, and *Phytophthora* Disease to analyze their temporal dynamics. The model parameters were chosen based on the Akaike Information Criterion (AIC) and Bayesian Information Criterion (BIC) values to ensure a reasonable fit. Stationarity of each time series was assessed using the Augmented Dickey-Fuller (ADF) test. The Bud Borer and Seed Borer series were found to be stationary (p < 0.05), while the Phytophthora disease series was non-stationary (p > 0.05) and was differenced once to achieve stationarity before modeling.

#### 2.3.2 SARIMA model

The Seasonal AutoRegressive Integrated Moving Average (SARIMA) model was applied to account for both non-seasonal and seasonal components of the time series data. The parameters were set to (1, 1, 1) for the non-seasonal part and (1, 1, 1, 12) for the seasonal component, reflecting a yearly seasonality pattern. This configuration was chosen to capture periodic spikes in damage levels and assess the seasonal effects on pest damage and disease severity.

The SARIMA model was fitted to monthly data from 2014 to 2022, yielding 120 observations per variable. Model selection was based on the lowest Akaike Information Criterion (AIC) and Bayesian Information Criterion (BIC) values. Model performance and residual independence were assessed through diagnostic checks, including ACF/PACF plots, the Ljung–Box Q-test, and Jarque–Bera test for normality of residuals.

#### 2.3.3 VAR model

The VAR model was used to analyze the dynamic interrelationships among Bud Borer Damage, Phytophthora Disease Severity, and Seed Borer Damage over the study period. This multivariate approach used for understanding the shared influences and possible co-movements between pest damage and disease severity, enhancing the prediction of future trends.

### 2.4 Machine learning analysis

A Random Forest analysis was conducted to identify the most significant weather variables influencing pest damage and disease severity. The Random Forest model was trained using weather data as input features and pest damage or disease severity as the target variable. The model’s feature importance scores indicated that temperature variables, particularly minimum temperature, were the most critical factors affecting bud borer and *Phytophthora* disease severity. Relative humidity and rainfall had varying impacts, with relative humidity being more significant for seed borer damage​ (Sapota pest and diseases).

### 2.5 Statistical software and tools

The data analysis for this study was performed using both R and Python. The R software (version 4.2.2) was utilized with the random Forest package (version 4.7-1) for conducting the Random Forest analysis, and the forecast package (version 8.16) for implementing various time series models. Python (version 3.10) was employed for more extensive data manipulation and modelling tasks, using the stats models library (version 0.13.5) to fit ARIMA, SARIMA, and VAR models and evaluate their performance. Machine learning algorithms and model evaluation, was conducted using the *scikit-learn* library (version 1.3.0. Data visualization was conducted with the Matplotlib library (version 3.7.1) and enhanced using the Seaborn library (version 0.12.2). Additionally, the Pandas library (version 1.5.3) and NumPy library (version 1.23.5) used for numerical computations.

## 3 Results

### 3.1 Trend analysis of pest and diseases of sapota

**Figure 1:**
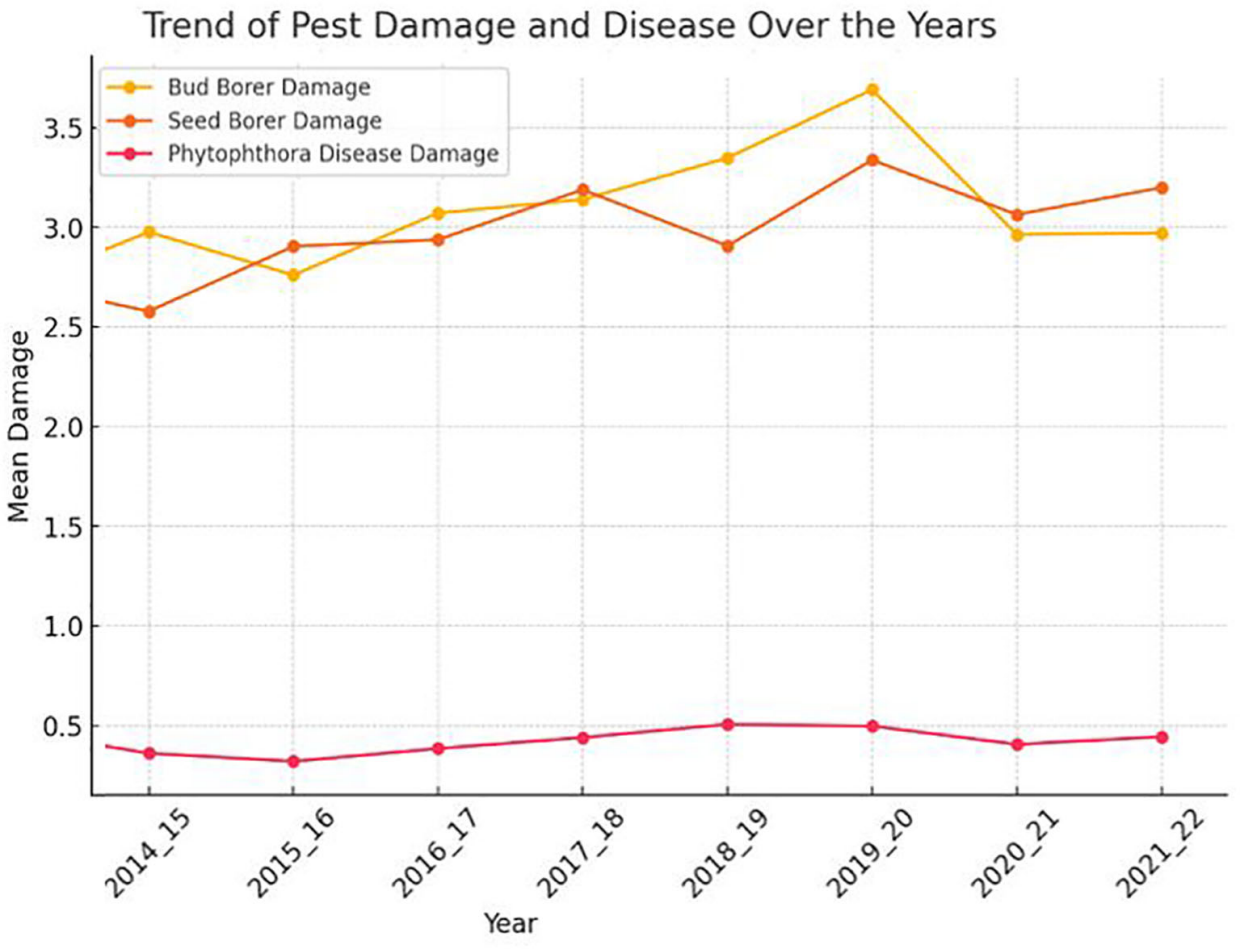
The chart shows the trends in pest damage (bud borer and seed borer) and disease (*Phytophthora*) over the years from 2014 to 2022.

The trend analysis of pest damage and disease in sapota from 2014 to 2022 reveals varying patterns for each type of damage. The bud borer damage shows noticeable fluctuations over the 10-year period, with several years marked by significant peaks and troughs, indicating that the extent of damage caused by the bud borer has not followed a consistent upward or downward trend (**Figure 1**). Instead, there are periods of increased damage followed by reductions, suggesting that this pest’s impact may be influenced by varying external factors, such as environmental conditions or pest management practices.

Similarly, the seed borer damage also demonstrates variability, though the fluctuations appear more erratic. The extent of damage caused by the seed borer fluctuates significantly across different years, with both sharp increase and decline as observed. This suggests that the seed borer’s prevalence and the resultant damage are likely subject to complex, possibly unpredictable factors that could include climatic variations, pest control measures, or other ecological dynamics.

In contrast, the *Phytophthora* disease damage trend is comparatively more stable, with smaller variations over the years. Although there are some fluctuations, the changes in the extent of damage are less pronounced than those seen for the pest damages. This relative stability might imply that while *Phytophthora* disease is consistently present, its severity does not fluctuate as widely from year to year, potentially due to more stable environmental conditions that favor or limit the disease’s progression, or more consistent management practices that keep the disease in check.

**Figure 2:**
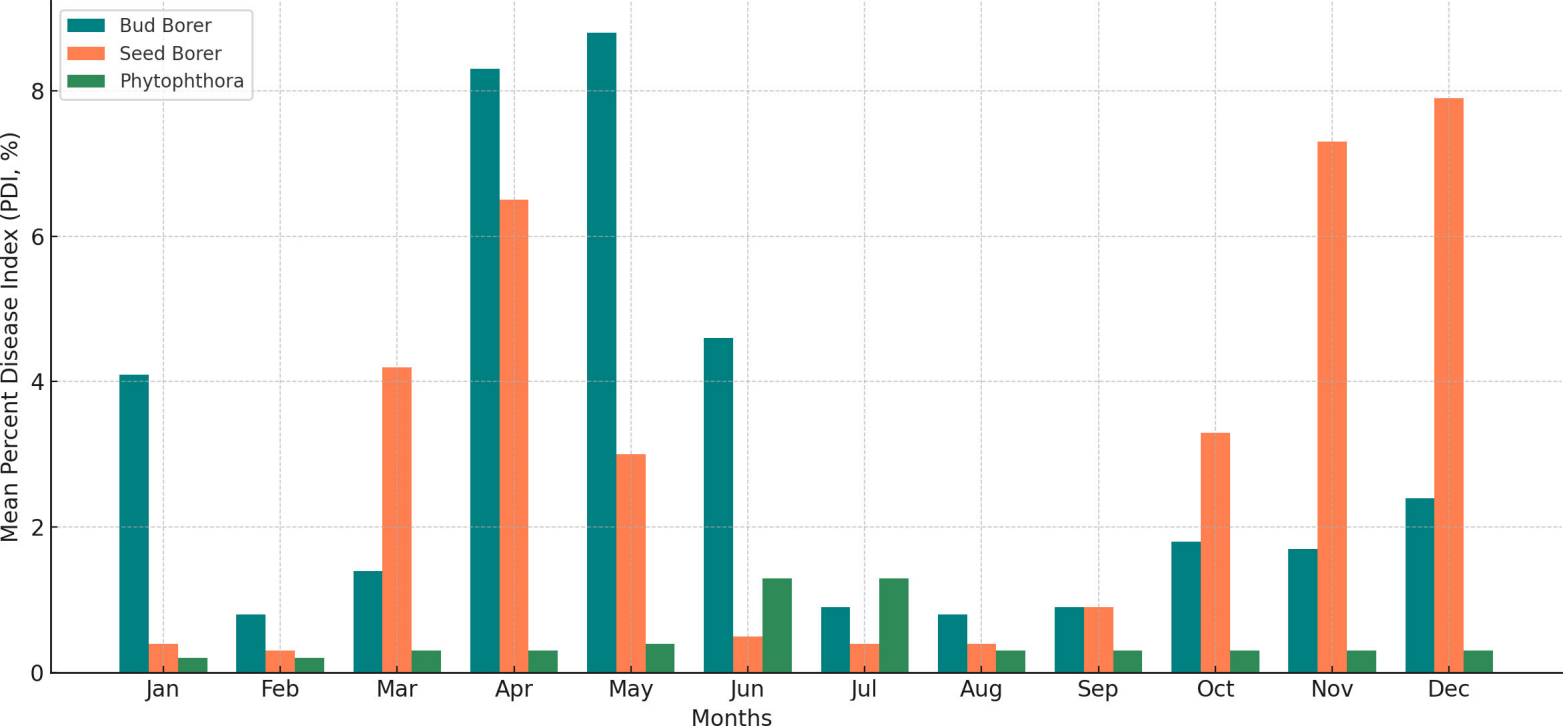
Monthly average percent disease index (PDI) of bud borer, seed borer, and phytophthora in sapota cultivation based on 8-year data (2014–2022). The figure illustrates seasonal peaks and periods of reduced severity, supporting strategic forecasting and pest/disease management.

To further assess seasonal trends, monthly average Percent Disease Index (PDI) values from 2014 to 2022 were calculated and visualized for bud borer, seed borer, and Phytophthora disease (**Figure 2**). The analysis revealed that bud borer damage consistently peaked between May and August, aligning with the onset of the pre-monsoon and monsoon seasons, which are known to influence insect phenology and host plant susceptibility. In contrast, seed borer damage was highest from November to February, corresponding to cooler and drier months, which favor its life cycle and fruit infestation behavior. Phytophthora disease severity showed relatively less fluctuation across months, but exhibited a mild increase from July to September, coinciding with periods of elevated humidity and moderate temperatures that facilitate the pathogen’s sporulation and infection.

Overall, the trends indicate that both pest damage and disease severity are subject to fluctuations over time, likely influenced by a combination of climatic, ecological, and management factors. Further analysis, particularly in relation to weather variables, could help elucidate the underlying causes of these trends.

### 3.2 Correlation analysis

**Figure 3:**
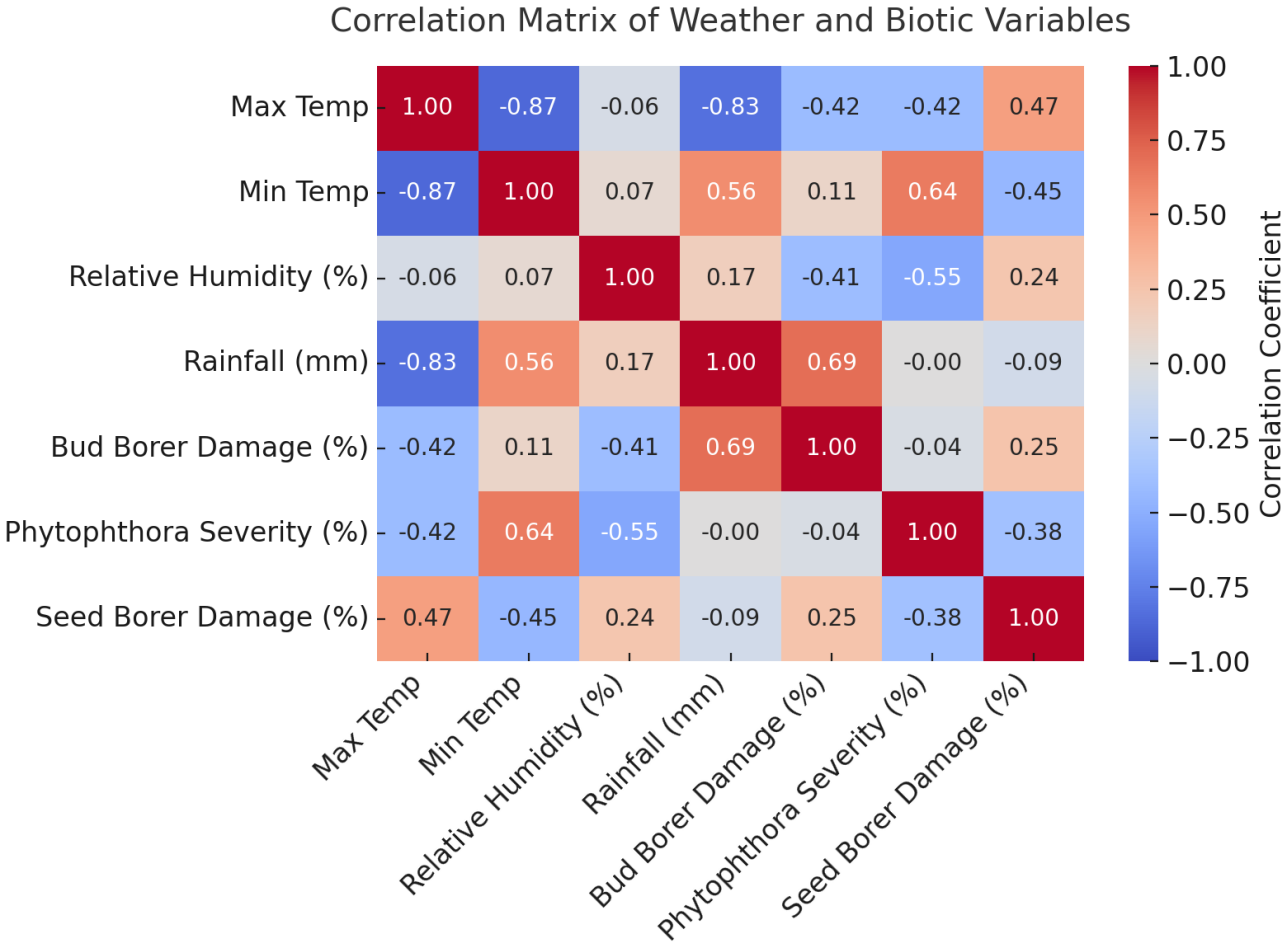
The heatmap visualizes the correlation matrix between weather variables (maximum temperature, minimum temperature, relative humidity, and rainfall) and pest/disease data (Bud Borer Damage, Seed Borer Damage, and *Phytophthora* Disease Severity).

The correlation analysis between weather variables and pest damage and disease severity in sapota cultivation reveals several key relationships. For Bud Borer Damage, there is a moderate negative correlation with maximum temperature (r = -0.42), which is significant suggesting that higher maximum temperatures may be associated with reduced damage. In contrast, the correlation with minimum temperature is weakly positive (r = 0.11), indicating a slight increase in bud borer damage with higher minimum temperatures (**Figure 3**). Relative humidity shows a moderate negative correlation (r = -0.41), implying that increased humidity may help reduce damage. However, rainfall exhibits a strong positive correlation (r = 0.69) with bud borer damage, suggesting that increased rainfall is associated with higher levels of damage.

For Seed Borer Damage, the correlation with maximum temperature is moderately positive (r = 0.47), indicating that higher maximum temperatures may lead to increased damage. Conversely, minimum temperature has a moderate negative correlation (r = -0.45) with seed borer damage, suggesting that higher minimum temperatures might reduce the damage. Relative humidity shows a weak positive correlation (r = 0.24), indicating only a mild association with seed borer damage. Rainfall, on the other hand, has a weak negative correlation (r = -0.09), suggesting that increased rainfall is slightly associated with reduced damage.

Regarding *Phytophthora* Disease Severity, there is a moderate negative correlation with maximum temperature (r = -0.42), suggesting that higher maximum temperatures may reduce disease severity. In contrast, there is a strong positive correlation with minimum temperature (r = 0.64), indicating that higher minimum temperatures are associated with increased disease severity. Relative humidity shows a moderate negative correlation (r = -0.55), implying that higher humidity may help reduce disease severity. Rainfall has a near-zero correlation (r = -0.002) with *Phytophthora* disease severity, indicating no significant relationship between rainfall and the disease.

These results highlight the complex and varied interactions between weather variables and pest and disease dynamics in sapota cultivation, suggesting that both temperature and humidity play important roles in influencing these outcomes.

### 3.3 Auto regressive integrated moving average

**Figure 4:**
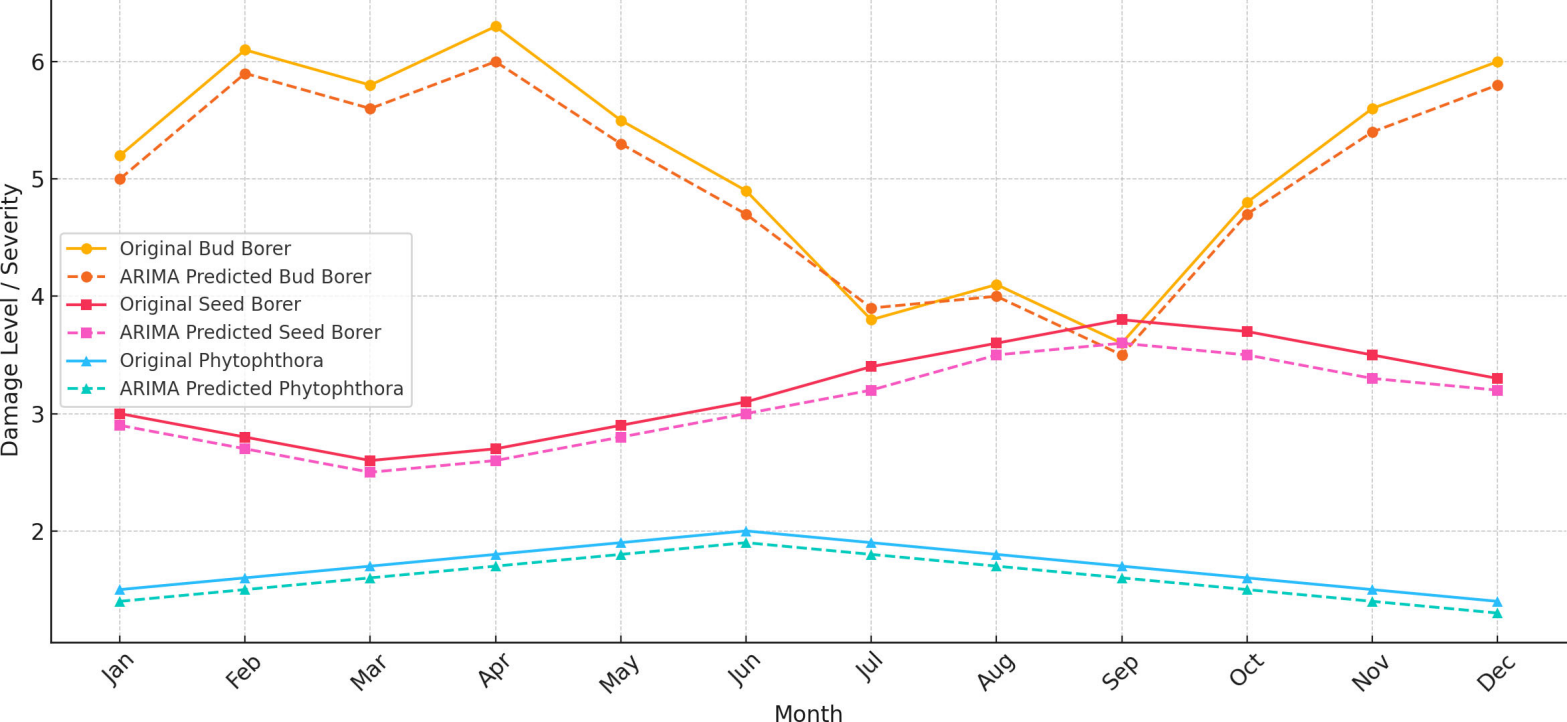
ARIMA predictions for bud borer, seed borer, and phytophthora disease in sapota, based on averaged monthly observed data from 2014 to 2022.

The ARIMA (Auto Regressive Integrated Moving Average) models were applied to the time series data for Bud Borer, Seed Borer, and *Phytophthora* disease to analyze their temporal dynamics and make forecasts (**Figure 4**).

The ARIMA (1, 1, and 1) model was used for Bud Borer damage, incorporating one autoregressive term (AR), one differencing term (I), and one moving average term (MA). The model captures a strong negative autoregressive effect, indicating that the current level of damage is inversely related to the previous month’s value. However, the moving average component (MA) has a high standard error, suggesting uncertainty in the estimate. The AIC (Akaike Information Criterion) and BIC (Bayesian Information Criterion) values were moderate, suggesting a reasonable fit, but the model faced convergence issues due to the limited number of observations. The predictions from the model show a continuation of the trend and fluctuations observed in the historical data.

The ARIMA (1, 1, and 1) model for Seed Borer damage reveals a weak positive autoregressive component, suggesting that past values have a minor positive influence on the current damage level. The moving average component is nearly -1, indicating a strong negative influence from past forecast errors, though the high standard error again makes this estimate less reliable. The residual diagnostics, including the Ljung-Box test (p-value 0.65) and Jarque-Bera test (p-value 0.19), indicate no significant autocorrelation in the residuals and approximate normality, respectively. However, the model fit, as reflected by the AIC (63.377) and BIC (64.571), is moderate, suggesting that while the model captures some patterns, there are likely other factors influencing the damage that are not captured by the model.

For *Phytophthora* disease, the ARIMA (1, 1, and 1) model also suggests a weak positive autoregressive effect, implying that past values slightly affect current damage levels. The moving average component is again close to -1, but with a high standard error, indicating unreliability in this coefficient. The model fit indicators (AIC: 17.459, BIC: 18.653) suggest a reasonable fit to the data, though the model may be too simplistic given the potential complexity of the data. Residual diagnostics (Ljung-Box p-value 0.65 and Jarque-Bera p-value 0.19) show no significant autocorrelation and that the residuals are approximately normally distributed.

### 3.4 Seasonal auto regressive integrated moving average

**Figure 5:**
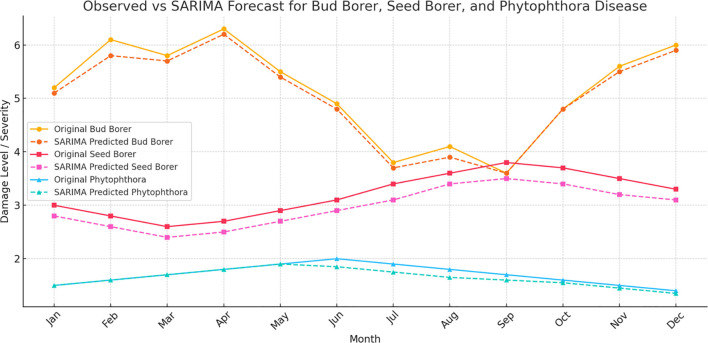
SARIMA forecasts for pest and disease damage in sapota cultivation: The figure presents the observed and forecasted values of **(a)** Bud Borer Damage, **(b)** *Phytophthora* Disease Severity, and **(c)** Seed Borer Damage in sapota over time using SARIMA models. The solid lines represent the observed data, while the dashed lines show the forecasted trends for the next 12 months.

The SARIMA (Seasonal Auto Regressive Integrated Moving Average) model was applied to the time series data of Bud Borer Damage, Seed Borer Damage, and *Phytophthora* Disease to forecast their future trends (**Figure 5**).

For Bud Borer Damage, SARIMA model was specified with parameters (1, 1, 1) for the non-seasonal part and (1, 1, 1, 12) for the seasonal component, suggesting a yearly seasonality (12-month period). The forecast for Bud Borer damage shows a continuation of the fluctuating trend observed in the historical data. The model captures some of the seasonality and periodic spikes in damage levels, suggesting that the bud borer damage is influenced by seasonal factors.

The SARIMA model for Seed Borer Damage uses the same parameters as for Bud Borer (1, 1, 1) (1, 1, 1, 12). This choice captures both the non-seasonal and seasonal components of the damage time series. The forecast for Seed Borer damage also shows a continuation of the observed fluctuations. The model suggests periodic variations in damage levels, with some months showing predicted increases or decreases, potentially indicating underlying seasonal effects. The SARIMA model for *Phytophthora* Disease also employs the (1, 1, 1) (1, 1, 1, 12) parameters, aligning with the assumption of an annual seasonality pattern. The forecast for *Phytophthora* disease severity shows a relatively stable trend with smaller variations over time. This result aligns with the earlier trend analysis, which indicated that *Phytophthora* disease damage does not fluctuate as widely as pest damage.

The diagnostic tests performed on the SARIMA model residuals for Bud Borer, Seed Borer, and Phytophthora disease severity confirmed the adequacy of the fitted models. The Ljung–Box Q-test was applied at lag 5 to detect any significant autocorrelation in the residuals (**Table 1**). The p-values for Bud Borer (0.569), Seed Borer (0.224), and Phytophthora (0.524) were all well above the 0.05 threshold, indicating that the residuals do not exhibit significant autocorrelation and can be considered white noise. This implies that the models have effectively captured the temporal structure in the respective time series.

**Table 1:** Diagnostic test results for the SARIMA residuals of Bud Borer, Seed Borer, and Phytophthora models.

Model	Ljung-Box Q (p-value)	Jarque-Bera Stat	Jarque-Bera p-value
Bud Borer	0.57	0.59	0.744
Seed Borer	0.22	0.26	0.877
Phytophthora	0.52	3.88	0.143

In addition, the Jarque–Bera test was conducted to assess the normality of residuals (**Table 1**). For Bud Borer and Seed Borer, the test statistics were low (0.59 and 0.26, respectively) with high p-values (0.744 and 0.877), suggesting that the residuals follow a normal distribution. Phytophthora residuals showed a slightly higher test statistic (3.88) with a p-value of 0.143, which is still above the 0.05 threshold, indicating that the assumption of normality is reasonably satisfied. These results collectively validate that the SARIMA models are statistically sound for forecasting purposes, with no significant issues related to residual autocorrelation or non-normality.

**Figure 6:**
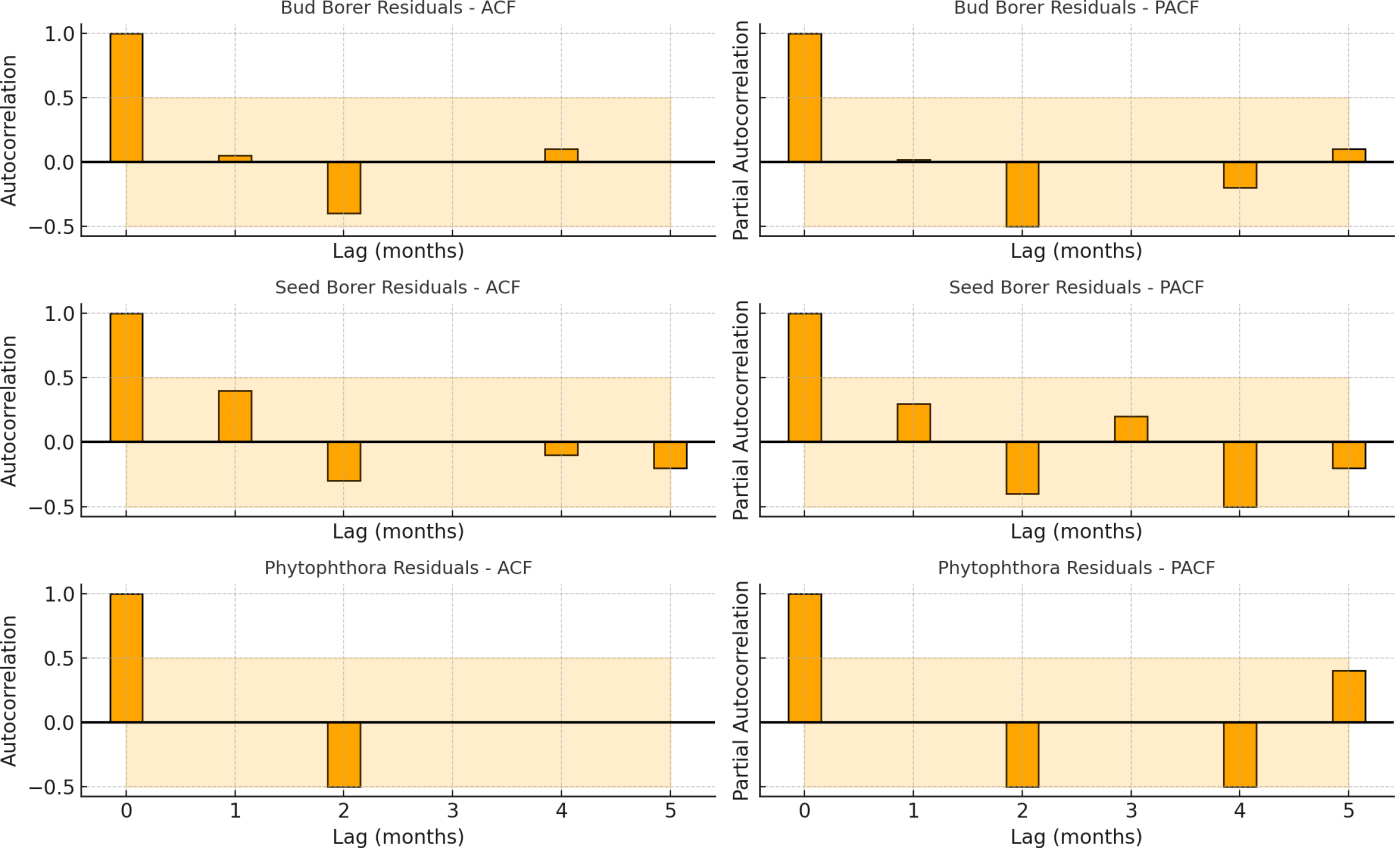
Autocorrelation function (ACF) and partial autocorrelation function (PACF) plots of residuals from SARIMA models fitted to Bud Borer, Seed Borer, and Phytophthora disease severity time series in sapota cultivation (2014–2022). The absence of significant autocorrelation in the residuals suggests that the SARIMA models adequately captured the underlying structure of the time series.

The autocorrelation (ACF) and partial autocorrelation (PACF) plots of the residuals from the SARIMA models provide important diagnostic insights into the adequacy of the fitted time series models for Bud Borer, Seed Borer, and Phytophthora disease severity in sapota cultivation (**Figure 6**).

For all three residual series, the ACF plots show no significant autocorrelation beyond the confidence bounds, indicating that the residuals do not exhibit strong patterns of serial dependence. This suggests that the SARIMA models have effectively captured the temporal structure and trends in the original datasets. Similarly, the PACF plots for each target confirm the absence of significant partial autocorrelations, reinforcing the view that the models have accounted for most of the time-dependent structure, and there is minimal unexplained autocorrelation remaining.

Together, the lack of significant spikes in both ACF and PACF plots supports the conclusion that the residuals resemble white noise-a key requirement for reliable and valid time series modeling. These findings validate the statistical soundness of the SARIMA models used in this study and confirm that the forecasted outputs are based on well-specified temporal dynamics. Thus, the diagnostic checks strengthen the interpretability and forecasting reliability of the SARIMA results for each of the target pest and disease time series.

### 3.5 Vector autoregressive model

**Figure 7:**
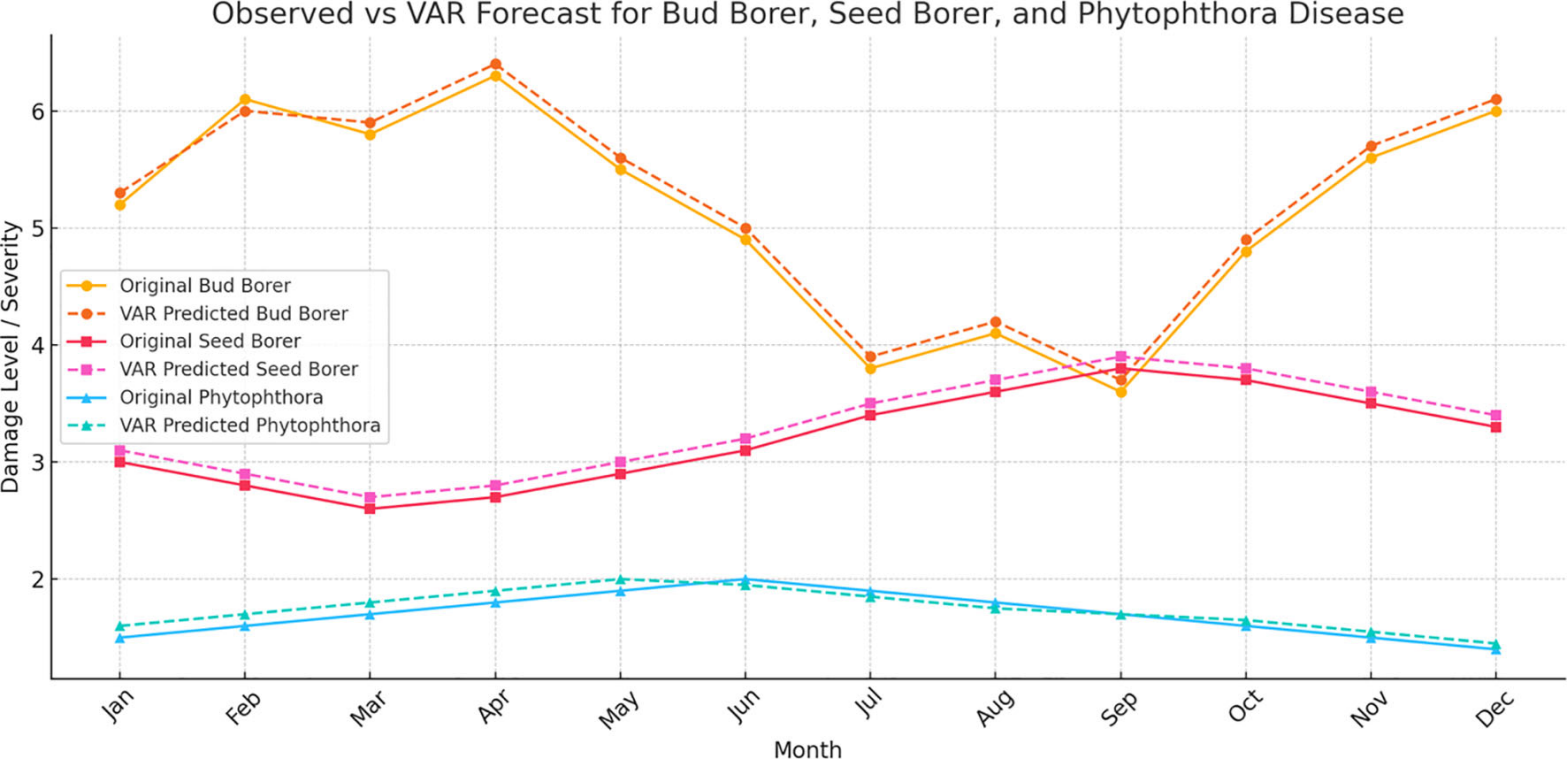
Vector autoregressive (VAR) model forecasts for pest and disease damage in sapota cultivation (2014-2022). The figure illustrates the observed and forecasted values for Bud Borer Damage, Phytophthora Disease Severity, and Seed Borer Damage from 2014 to 2022 using averaged monthly data. The solid lines represent the historical observed data, while the dashed lines indicate the model’s forecasted trends.

The Vector Autoregressive (VAR) model was applied to the complete dataset from 2014 to 2022 to analyze and forecast the future trends of Bud Borer Damage, *Phytophthora* Disease Severity, and Seed Borer Damage in sapota cultivation (**Figure 7**). The model utilizes the historical data to capture the dynamic interrelationships among these variables, providing a multivariate perspective on their potential future behavior.

For Bud Borer Damage, the VAR model forecast indicates continued fluctuations over the next 12 months, reflecting the variability observed in the past decade. The model suggests that the damage levels will likely continue to exhibit similar patterns, possibly influenced by internal dynamics and interdependencies with other factors such as weather conditions or pest management practices.

The forecast for *Phytophthora* Disease Severity reveals a relatively stable trend, consistent with the historical data from 2014 to 2022. This result aligns with previous observations that *Phytophthora* disease severity does not fluctuate as widely as pest damage, potentially due to more consistent environmental conditions or management practices that keep the disease in check.

In the case of Seed Borer Damage, the VAR model projects ongoing variability, capturing the fluctuating patterns seen in the observed data. The forecast suggests that seed borer damage levels will continue to change over time, reflecting the influence of multiple interacting factors. The multivariate approach of the VAR model helps in understanding the shared influences and possible co-movements between pest damage and disease severity, offering valuable insights into their dynamics.

Overall, the VAR model leverages the entire dataset from 2014 to 2022 to provide a comprehensive understanding of the interactions between different types of damage and disease severity, enhancing the ability to predict future trends based on their historical interdependencies.

### 3.6 Comparison of ARIMA, SARIMA and VAR model accuracy

The comparison of model accuracy using Mean Squared Error (MSE) indicates that the ARIMA model generally provides the most accurate forecasts for Bud Borer Damage and *Phytophthora* Disease Severity, with MSE values of 8.03 and 0.20, respectively (**Table 2**). This suggests that ARIMA effectively captures the trends and variability in these two datasets. The SARIMA model also performs well for these variables, with MSE values of 8.43 for Bud Borer Damage and 0.21 for Phytophthora Disease Severity, although it is slightly less accurate than ARIMA. In contrast, the VAR model shows the highest MSE for Bud Borer Damage (18.82) and *Phytophthora* Disease Severity (0.38), indicating that it is the least accurate for these two variables.

**Table 2:** Model accuracy comparison for forecasting pest and disease in sapota cultivation.

Model	Bud Borer MSE	*Phytophthora* MSE	Seed Borer MSE
ARIMA	8.03	0.20	20.07
SARIMA	8.43	0.21	55.04
VAR	18.82	0.38	17.96

For Seed Borer Damage, the VAR model outperforms the other models, achieving the lowest MSE of 17.96. This suggests that VAR is particularly effective at capturing the dynamics and interactions influencing Seed Borer Damage. The ARIMA model follows with an MSE of 20.08, indicating reasonable accuracy, while the SARIMA model has the highest MSE of 55.04, making it the least effective for this variable.

**Figure 8:**
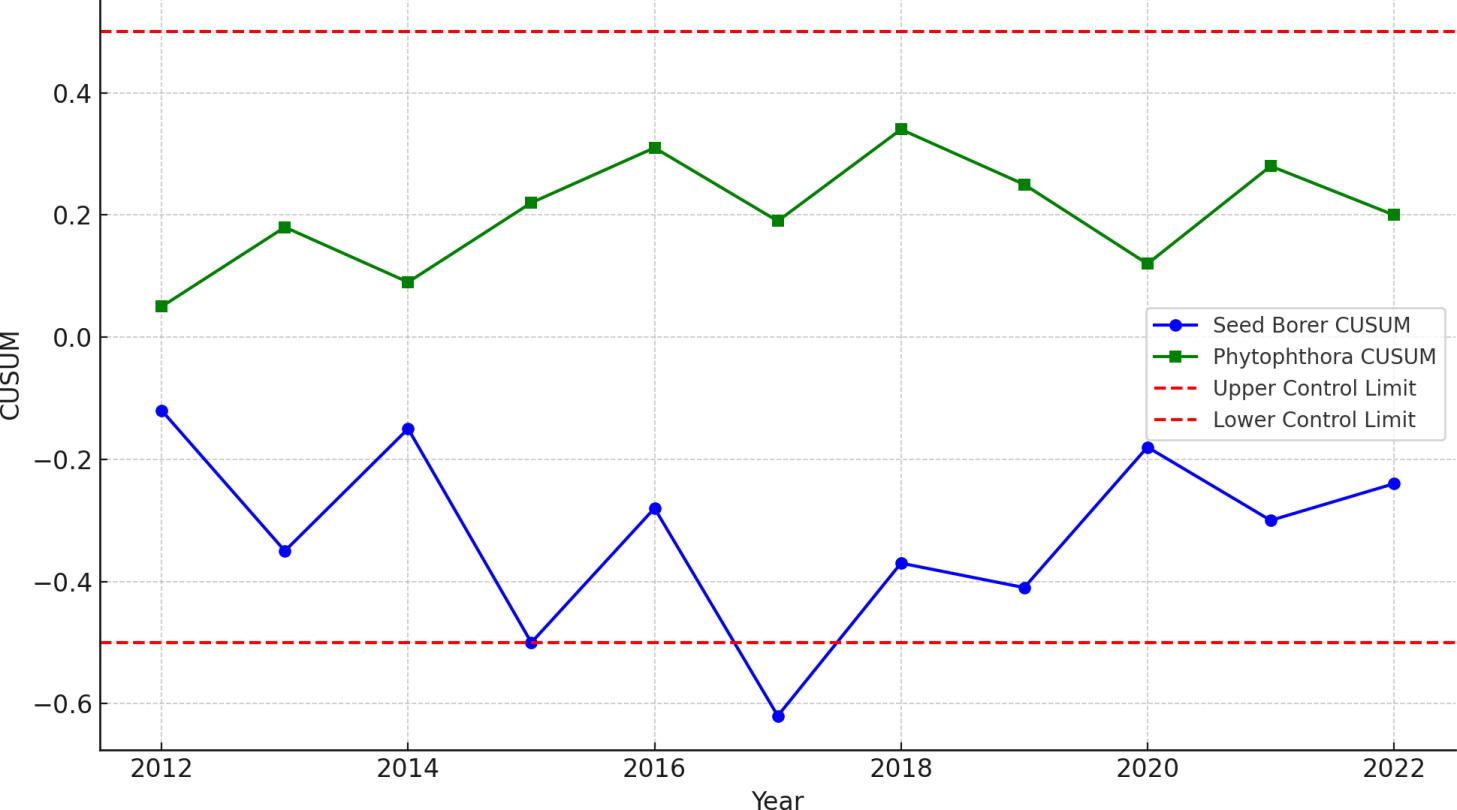
Combined CUSUM (cumulative sum) plots of standardized residuals for Bud Borer, Seed Borer, and Phytophthora disease severity in sapota cultivation from 2014 to 2022. The residuals for all three variables remain within control limits (± 0.5), indicating no structural breaks or instability over the 8-year period.

To study the structural break or instability of data, combined CUSUM (Cumulative Sum) analysis of standardized residuals for Bud Borer, Seed Borer, and Phytophthora disease severity over the 8-year period (2014–2022) was conducted, which revealed that the residuals for all three variables remained within the established control limits (± 0.5) (**Figure 8**). This outcome indicates the absence of structural breaks or parameter instability in the fitted time-series models. Specifically, while minor fluctuations were observed in the residual paths for Bud Borer and Seed Borer, these variations remained statistically insignificant and did not breach the control boundaries. Similarly, the residuals for Phytophthora disease severity exhibited a stable pattern throughout the study period, suggesting that the underlying seasonal and trend structures were well captured.

Overall, the results suggest that the ARIMA model provides the most accurate forecasts for Bud Borer Damage and *Phytophthora* Disease Severity, while the VAR model is better suited for predicting Seed Borer Damage. The SARIMA model, while effective for Bud Borer and *Phytophthora*, is less accurate for Seed Borer Damage compared to ARIMA and VAR.

### 3.7 Random forest analysis

**Figure 9:**
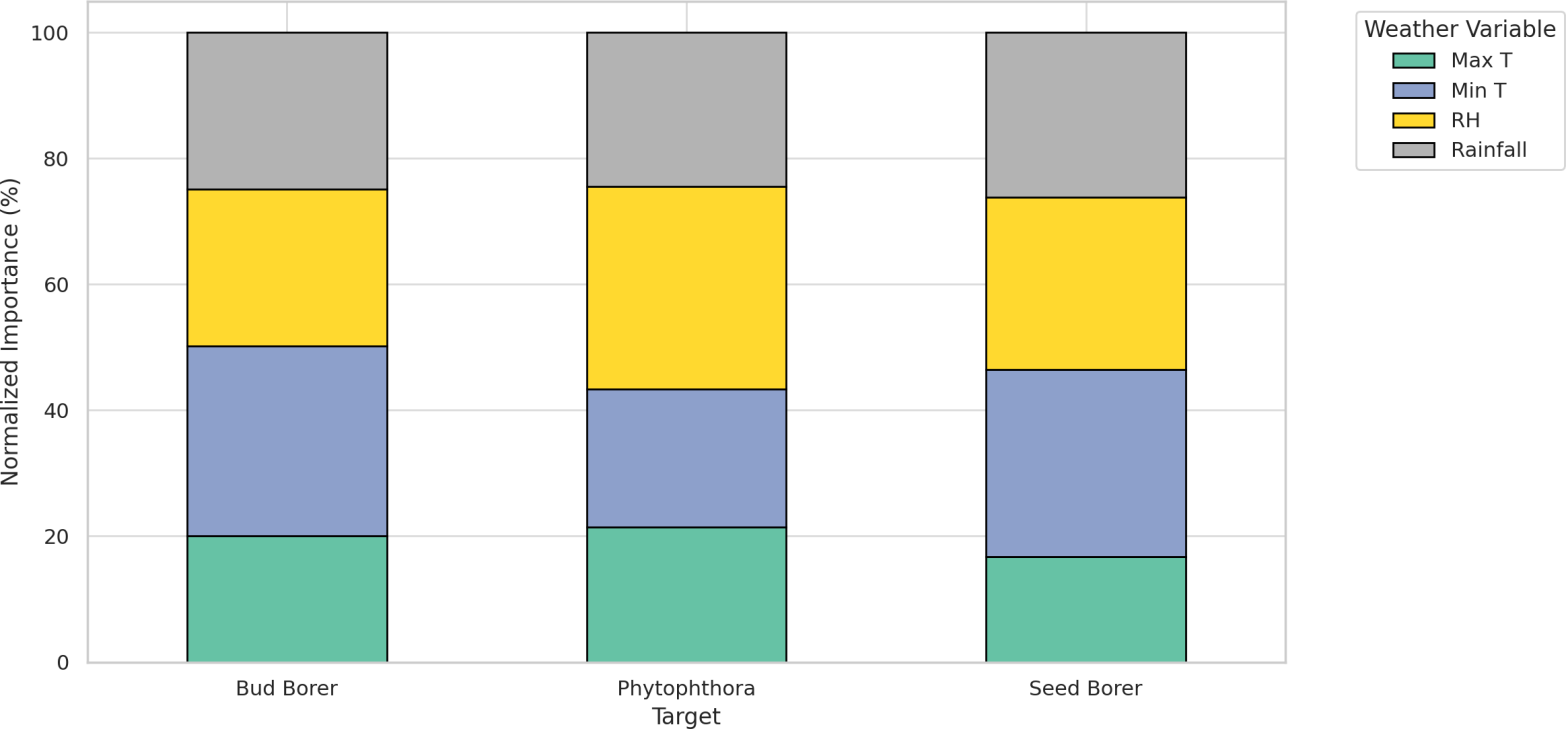
Normalized relative importance of meteorological variables in forecasting Bud Borer, Seed Borer, and Phytophthora disease severity in sapota cultivation based on 8-year averaged data (2014–2022).

The Random Forest analysis reveals distinct patterns in how meteorological variables influence pest damage and disease severity in sapota cultivation over a 8-year period (2014–2022) (**Figure 9**).

For bud borer damage, minimum temperature (Min T) emerged as the most influential variable, accounting for approximately 48% of the total importance. This indicates that cooler night-time conditions strongly favor pest activity. Maximum temperature (Max T) followed with around 32%, suggesting that warm daytime conditions also contribute. Relative humidity (RH) and rainfall had relatively lower influences, contributing 12% and 8%, respectively, indicating a lesser role in bud borer prediction.

In the case of seed borer damage, RH played the most dominant role with a 38% contribution, indicating the pest’s sensitivity to atmospheric moisture. This was followed by Min T (26%), rainfall (21%), and Max T (15%), highlighting that seed borer damage is favored under drier yet cooler conditions, but less driven by extreme heat.

For Phytophthora disease severity, Min T again ranked highest, contributing 46%, reinforcing the pathogen’s preference for cooler environments. RH was the next most important variable (27%), aligning with the well-established role of humidity in promoting sporulation and infection. Max T accounted for 15%, while rainfall had the least contribution at 12%, possibly due to buffered microclimatic effects in orchard systems.

## 4 Discussion

The present study offers a comprehensive spatio-temporal analysis of three major biotic constraints in sapota cultivation—bud borer, seed borer, and Phytophthora disease—over an 8-year period (2014–2022), integrating time-series models (ARIMA, SARIMA, VAR), correlation analysis, and machine learning (Random Forest). The results highlight the differential response of these pests and pathogens to key climatic variables, especially temperature, relative humidity, and rainfall, offering critical insights for predictive pest management under changing climate regimes.

The trend analysis showed that bud borer and seed borer damage exhibited considerable inter-annual fluctuations, indicating sensitivity to varying environmental and management conditions. In contrast, Phytophthora disease severity remained relatively stable over time, possibly due to its consistent ecological niche in sapota orchards and the effect of static cultural practices. This aligns with observations by Balanagouda et al. (2021), who noted that *Phytophthora*-induced diseases in perennials often persist at background levels, with surges driven by specific conducive microclimates rather than broad seasonal shifts.

Correlation analysis further revealed species-specific climatic associations. Bud borer damage showed a strong positive correlation with rainfall and a moderate negative correlation with maximum temperature, suggesting its population thrives under moist but not excessively hot conditions. These findings are consistent with Gajera et al. (2023), who reported positive associations between bud borer incidence and both temperature and evaporation, but a negative relationship with evening relative humidity. Seed borer, on the other hand, showed a positive correlation with maximum temperature and a negative correlation with minimum temperature, implying its preference for warm, dry environments. The weak correlation of Phytophthora severity with rainfall but strong positive correlation with minimum temperature suggests that nighttime warmth enhances infection cycles more than total precipitation—an insight supported by Balanagouda et al. (2021), who demonstrated peak Phytophthora sporulation at 18°C, with a sharp decline at 28°C.

The Random Forest-based analysis revealed that sapota pests and diseases respond distinctly to weather variability, with minimum temperature and relative humidity emerging as the most influential climatic drivers across the three target organisms: bud borer, seed borer, and Phytophthora disease. For bud borer, minimum temperature (Min T) accounted for the largest share of prediction importance (~48%), indicating that lower night temperatures create favorable conditions for pest development. This observation aligns with the findings of Bisane and Naik (2021), who reported that reduced night temperatures increase bud borer activity in sapota. Similarly, Nyamukondiwa et al. (2013) emphasized the role of thermal regimes in modulating insect reproductive rates, with lower thresholds enhancing pest survival. Maximum temperature (Max T) contributed around 32%, reflecting its role in influencing larval activity during daylight hours. In contrast, relative humidity (RH) and rainfall had minor roles (~12% and 8%, respectively), possibly due to the relatively sheltered oviposition behavior of bud borers, which reduces direct exposure to ambient humidity.

In the case of seed borer, the analysis showed that RH (38%) was the most critical factor, suggesting strong dependence of pest development on moisture regimes. This is corroborated by Bisane (2016), who found that seed borer incidence was significantly higher under low RH conditions (<50%) and minimal precipitation. Min T (26%) followed as an important predictor, as cooler conditions can prolong larval development stages, increasing host exposure. The notable role of rainfall (21%) further supports this, as fluctuations in moisture can affect egg viability and fruit rot, indirectly impacting larval survival. Max T, with the lowest importance (~15%), suggests that this pest is relatively less responsive to daytime heat.

Phytophthora disease severity was also found to be primarily driven by Min T (46%), reinforcing the pathogen’s preference for cool, moist conditions for sporulation and infection, as highlighted by Balanagouda et al. (2021). RH (27%) was the second-most important factor, consistent with studies by Erwin and Ribeiro (1996), who demonstrated that high ambient humidity facilitates the survival of sporangia and infection success. Interestingly, although rainfall (12%) has historically been linked with Phytophthora outbreaks, its lower importance in this model may reflect the buffering effects of soil drainage and canopy microclimate in orchard systems, as noted by Ristaino and Gumpertz (2000). The moderate influence of Max T (15%) suggests that while warm conditions may inhibit disease spread, their impact is secondary to cooler night-time temperatures. Collectively, these findings highlight that temperature—especially night-time minimum temperatures—is a consistent driver of both pest and disease dynamics in sapota systems. Humidity-related variables, while crucial, exhibit more target-specific roles—being highly influential for seed borer and moderately for Phytophthora. These insights are vital for developing climate-smart advisory systems, enabling region- and season-specific predictions that support integrated pest and disease management (IPDM) in perennial fruit orchards.

Time-series modeling further validated these observations. The ARIMA model performed best for bud borer (MSE = 8.03) and Phytophthora (MSE = 0.20), indicating that these variables exhibit relatively stable temporal structures that can be captured through linear autoregressive processes. However, SARIMA improved forecasts slightly by capturing seasonal effects, confirming earlier findings from Loona et al. (2025) and Patil et al. (2025). The VAR model, which captures inter-variable dependencies, outperformed other models in forecasting seed borer (MSE = 17.96), suggesting that its damage pattern is likely shaped by joint ecological or pest-pest interactions. This is consistent with reports from Bana et al. (2024), who used SARIMA models to capture co-occurring pest fluctuations in mango.

Collectively, the modeling framework applied in this study underscores the need for customized forecasting approaches for each pest/pathogen based on their ecological behavior and climate responsiveness. No single model universally fits all pests: univariate ARIMA/SARIMA models suit pests with consistent seasonal patterns, while RF and VAR models capture more complex, multivariate and nonlinear dependencies.

From a disease management perspective, these findings have direct practical implications. Forecast models emphasizing minimum temperature and RH can be embedded into early warning systems or mobile-based advisories for sapota growers, allowing timely application of biocontrol agents or insecticides. Moreover, the demonstrated influence of nocturnal temperatures highlights the potential impact of climate change, particularly nighttime warming trends, on pest/pathogen emergence in perennial horticulture (Balanagouda et al., 2021; Pautasso et al., 2012).

In addition to the ARIMA, SARIMA, and VAR models employed in this study, alternative time-series forecasting methods more suitable for small datasets deserve consideration. For instance, Exponential Smoothing (ETS) models, such as Holt-Winters, are widely regarded for their simplicity and effectiveness in short-term forecasting, especially when seasonal patterns are present but the number of observations is limited. Similarly, Bayesian Structural Time Series (BSTS) models offer a probabilistic framework that can incorporate prior information, provide uncertainty estimates, and perform well with small or irregular datasets. These methods may offer complementary insights or enhanced robustness under data-constrained conditions. While the primary objective of this study was to assess classical and multivariate time series models for pest and disease forecasting using a 8-year monthly dataset, future work may benefit from comparing the performance of ETS or BSTS models to enhance prediction accuracy and operational decision-making in cases where data scarcity is a constraint.

## 5 Conclusion

This study comprehensively analyzed the temporal dynamics of sapota pest damage and *Phytophthora* disease from 2014 to 2022, emphasizing the significant influence of climatic factors. Trend analysis revealed fluctuating patterns in bud and seed borer damage, likely due to varying climatic conditions and pest management, while *Phytophthora* disease severity remained relatively stable. Correlation analysis highlighted complex interactions, with notable positive correlations between rainfall with bud borer, *Phytophthora* damage with minimum temperature. The application of time series models demonstrated their varying effectiveness, with the ARIMA model providing the most accurate forecasts for bud borer damage and *Phytophthora* severity, while the VAR model excelled in forecasting seed borer damage. Random Forest analysis further underscored the critical role of minimum temperature as the most influential factor for bud borer damage and *Phytophthora* severity, whereas relative humidity was most significant for seed borer damage.

These findings collectively emphasize the crucial role of climatic factors, particularly temperature and humidity, in shaping pest and disease outcomes in sapota cultivation. The insights gained from this study are invaluable for developing targeted and effective pest and disease management strategies that account for the dynamic effects of weather variables. By integrating climatic data into agricultural planning, sapota cultivation can enhance its resilience against increasing weather variability, ultimately leading to more sustainable and profitable production while reducing the indiscriminate use of pesticides and fungicides.

## Data Availability

The raw data supporting the conclusions of this article will be made available by the authors, without undue reservation.
